# Unravelling the life history of Amazonian fishes through otolith microchemistry

**DOI:** 10.1098/rsos.160206

**Published:** 2016-06-08

**Authors:** Theodore W. Hermann, Donald J. Stewart, Karin E. Limburg, Leandro Castello

**Affiliations:** 1 Department of Environmental and Forest Biology, State University of New York, College of Environmental Science and Forestry, Syracuse, NY 13210, USA; 2 Department of Fish and Wildlife Conservation, Virginia Polytechnic Institute and State University, Blacksburg, VA, USA

**Keywords:** otolith microchemistry, Amazon, life history, fish migration

## Abstract

Amazonian fishes employ diverse migratory strategies, but the details of these behaviours remain poorly studied despite numerous environmental threats and heavy commercial exploitation of many species. Otolith microchemistry offers a practical, cost-effective means of studying fish life history in such a system. This study employed a multi-method, multi-elemental approach to elucidate the migrations of five Amazonian fishes: two ‘sedentary’ species (*Arapaima* sp. and *Plagioscion squamosissimus*), one ‘floodplain migrant’ (*Prochilodus nigricans*) and two long-distance migratory catfishes (*Brachyplatystoma rousseauxii* and *B. filamentosum*). The Sr : Ca and Zn : Ca patterns in *Arapaima* were consistent with its previously observed sedentary life history, whereas Sr : Ca and Mn : Ca indicated that *Plagioscion* may migrate among multiple, chemically distinct environments during different life-history stages. Mn : Ca was found to be potentially useful as a marker for identifying *Prochilodus*'s transition from its nursery habitats into black water. Sr : Ca and Ba : Ca suggested that *B. rousseauxii* resided in the Amazon estuary for the first 1.5–2 years of life, shown by the simultaneous increase/decrease of otolith Sr : Ca/Ba : Ca, respectively. Our results further suggested that *B. filamentosum* did not enter the estuary during its life history. These results introduce what should be a productive line of research desperately needed to better understand the migrations of these unique and imperilled fishes.

## 1. Introduction

Otoliths, the calcified ear-stone organs in fish hearing/balance systems, have been widely used to understand fish life history. Otolith incremental deposition forms the basis of age and growth studies [1–3], and incorporated elements and isotopes provide additional information on the life history of an individual [4–6]. Otoliths are metabolically inert, recording information over a fish's entire life [5]. Most studies on the chemical composition of otoliths have focused on coastal marine and diadromous fishes due to the great chemical disparity of the environments exploited by these fishes (e.g. river and estuarine versus marine environments). Otolith microchemistry studies (using micro- and trace elements) in freshwater fishes have elucidated natal origins [7–9], migration patterns [10–12] and pollution exposure history [13,14], providing critical information needed for the management of freshwater fisheries [15]. However, little work has been done on otolith microchemistry in tropical freshwater systems, even though they are under increasing anthropogenic pressures.

There is great need to study the life history of commercial fishes in the Amazon and other large South American basins, as there are dozens of exploited taxa with widely varying life histories that are poorly understood [16]. These basins provide ideal systems to study tropical fish otolith microchemistry. The Amazon Basin is geochemically diverse and complex, so many otolith chemical markers may be present. Despite the potential for otolith microchemistry studies, only five studies have used microchemical markers to understand fish migrations in the Amazon [17–21]. These studies used multicollector-inductively coupled plasma mass spectrometry (ICPMS), a powerful but expensive tool for quantifying isotope ratios, limited in the number of elements that can be sampled in one ablation pass. While Sr isotope ratios have proved to be useful in freshwater systems, it is well established that otoliths entrain many other useful micro- and trace chemicals in freshwaters, including manganese [22], bromine [23] and selenium [13], among others. An approach employing multiple elements to the study of fish otolith microchemistry could help elucidate the life histories and ecology of Amazonian fishes.

Low-resolution laser ablation–ICPMS (LA–ICPMS) and scanning X-ray fluorescence microscopy (SXFM), though unsuited for accurate quantification of multiple Sr isotopes, offer another cost-effective way to sample a multitude of elements simultaneously. The two methods complement one another in their ability to measure particular elements that the other cannot (e.g. LA–ICPMS can accurately measure Ba, while SXFM can measure Se) and may complement data provided by multicollector–ICPMS [24]. The flexibility to sample so many elements simultaneously is particularly important in poorly studied systems, like the Amazon, where elements that may be useful markers are largely unknown. Otolith microchemistry also provides a stark contrast to other methods of studying fish migration. Mark–recapture studies are prohibitively expensive in large or remote systems [25], while fish in tagging studies are often intensely harvested by fishermen (e.g. [26]) or migrate distances too great to study with these methods [27].

Here, we demonstrate the usefulness of a multi-element approach based on low-resolution LA–ICPMS and SXFM for unravelling the life histories of Amazonian commercial fishes.

### 1.1. Life histories of Amazonian fishes

The Amazon Basin is a complex network of interconnected terrestrial and aquatic environments, with chemical diversity dictated by the bedrocks and soils through which river tributaries pass [28]. The most abundant fish populations are in the várzea floodplains, which experience 10 m seasonal inundations by the sediment-laden and nutrient-rich whitewater rivers originating in the Andes Mountains [29,30]. When water levels rise, the main river and floodplains become hydrologically interconnected, prompting fishes to migrate out of lakes and river channels and onto floodplain forests and floating meadows (i.e. grasses) [31]. The flooded forests and floating meadows provide diverse and abundant food sources such as periphyton, insects and fruits, and they act as vital nursery habitats for the larvae and juveniles of many species [32–34]. As flood waters recede, most fishes migrate back to main river channels, where temperature and oxygen levels are more stable and tolerable, although many species adapted to warm and/or hypoxic conditions migrate to floodplain lakes and smaller channels during low waters where water quality is generally poor [35].

In the estuary of the Amazon, about 300 commercially important fish species [36] are supported by biological productivity that is driven not only by rising and receding floodwaters but also by tidal movements [37,38]. Some large migratory catfish species are hypothesized to use the estuary as nursery grounds [37], although a recent study suggests that the life histories of these fishes may be variable within and among related species [17].

Three main migratory strategies are recognized among Amazonian commercial fishes to maximize the spatial and temporal heterogeneity of resource availability. ‘Sedentary’ fishes generally live in floodplain lakes and migrate laterally through floodplain habitats following flood pulses, a behaviour documented in detail in the giant *Arapaima* sp. [39]. Sedentary fishes are typified by *Plagioscion squamosissimus*, a species common to floodplain lakes [40,41]. Studies of the larval ecology of *Plagioscion* indicate that they tolerate diverse water conditions, but prefer spawning in lentic areas with high temperature, low pH and low dissolved oxygen [42,43]. There is little work on the migration of adult individuals (but see [44,45]). It can be expected that *P. squamosissimus* migrates through floodplain habitats throughout the hydrological cycle like other sedentary fishes.

‘Floodplain migratory’ fishes are typified by various characoid fishes, such as *Prochilodus nigricans*. Available studies indicate that adult *Prochilodus* migrate upwards of several hundred kilometres into, or upstream in, the whitewater rivers of the Amazon mainstem (or major tributaries) as waters begin to rise [33,46]. Large schools broadcast spawn in these whitewater rivers, after which adults migrate laterally onto floodplain habitats to feed. Eggs and larvae drift downriver and settle in the flooded forest or floodplain meadows, where the developing juveniles feed, grow and, eventually, migrate back upstream at an age of about six months to complete the cycle [47]. By migrating between the nutrient-rich white water and nutrient-poor, acidic black waters, *Prochilodus* are exposed to more varied water chemistry than most other Amazonian fishes. However, the migration patterns of *Prochilodus* species and populations apparently vary in direction and timing in different Amazon tributaries or South American rivers (for summary, see [33]). The migratory ecology of *P. nigricans* in the Ecuadorian Amazon remains poorly studied. Silva & Stewart [48] found that growth parameters for *P. nigricans* in the Ecuadorian Amazon were inconsistent with those in Bolivia or Central Amazon (i.e. forming annuli versus biannuli in the latter two regions). They suggested a shorter hydrological cycle as a possible factor in the lower growth rates that they observed. This suggests that the time at which juveniles first migrate to rejoin adult populations might also differ among populations.

‘Long-distance catfish’ migrants are typified by several of the large pimelodid catfishes like *Brachyplatystoma rousseauxii* and *B. filamentosum*. Early studies indicated that adults migrate into the headwater rivers along the Andean foothills during rising waters, where they broadcast spawn [27,49,50]. Eggs, larvae and small juveniles drift downriver for thousands of kilometres, with some of them reaching the estuary. Here the small juveniles settle, feed and grow for several years before migrating up the Amazon mainstem towards the headwaters. The distance migrated during the complete cycle may total upwards of 8000 km [21]. However, the extent to which these fishes use the estuary as a nursery ground is unclear. Recent studies analysed strontium isotope ratios in otoliths and showed that these fishes have varied life histories, both within and among species [17,21]. Some individuals appear to home back to natal spawning grounds [21], indicating more complex life histories than previously thought.

Active research and funding for studying the life histories of Amazonian fishes are minimal, despite the commercial value of these fishes and the multiple environmental threats they face. Overfishing has led to considerable declines in some species [51], while deforestation is producing stronger, earlier flood pulses, altering the flood cycle that migratory fishes rely upon [52]. Hydroelectric damming, long considered a threat to Amazonian migratory fishes [37], is widespread, blocking migration routes and disrupting flood pulses [53,54]. Installation of ‘fish ladders’ to aid migratory fishes in circumventing these obstructions has been ineffective [55–58]. Pollution from gold mining [59], agriculture [60] and oil exploration [61] is damaging water quality, affecting the health of fish and humans [62–64]. Many of these species, particularly the migratory catfishes, traverse international boundaries during spawning migrations, which further complicates efforts to study their biology or implement effective management strategies. Otolith microchemistry not only reveals the entire recorded life history of these fishes, it also affords a means of doing it cost-effectively.

Here, we asked the following research questions regarding LA–ICPMS and SXFM. (i) Can these methods be used to identify (small-scale) migrations in sedentary fishes? (ii) Can they identify shifts between life-history stages in floodplain migrants? (iii) Can they identify habitat use in juvenile long-distance migrants? This study identified new chemical markers in the otoliths of five Amazonian fishes and unravelled new information on their life histories.

## 2. Material and methods

This study investigated the migratory behaviours of two sedentary species (*Arapaima* sp. and *P. squamosissimus*), one floodplain migrant (*P. nigricans*), and two long-distance migratory catfishes (*B. rousseauxii* and *B. filamentosum*) by analysing chemical patterns in their otoliths using low-resolution LA–ICPMS and SXFM. LA–ICPMS provided data collected along transects on the otoliths. This method can quantify barium, an important trace element in otolith studies [5], which cannot reliably be measured at low concentrations with SXFM due to measurement interference from a secondary calcium peak. SXFM provided data for elements not sampled by LA–ICPMS (such as selenium), as well as two-dimensional chemical ‘maps’ of entire otolith sections that reveal possible chemical heterogeneity within the otolith structure. We also analysed some otoliths using both methods to serve as a check for elements that can be quantified by both methods. Changes in measured elements over a fish's life were used to infer migration to and/or residency in various habitats.

### 2.1. Otolith collection and preparation

Fish otoliths were obtained from various locations. One otolith from *Arapaima* sp. (*n* = 1; 160 cm standard length (S.L.)) was collected in November, 2008 from Inkapati Head Pond near Apoteri, adjacent to the Essequibo River mainstream in Guyana (4°7.027′ N, 58°29.531′ W). Sagittal otoliths from *Plagioscion* (*n* = 1; length unknown) and lapillae from *Prochilodus* (*n* = 6; 97–267 mm S.L.) were collected in the Ecuadorian Amazon in April and May, 1999, from the lower reaches of the Cuyabeno drainage (0°15.50′ S, 75°53.97′ W), a blackwater river, near its confluence with the whitewater Rio Aguarico. A lapillus from *B. rousseauxii* (*n* = 1; 85 cm fork length (F.L.)) was also collected in April, 1999, near the confluence of the blackwater Rio Sabalo and Rio Aguarico (0°23.58′ S, 75°40.083′ W), Ecuador. Additional lapillae from *B. rousseauxii* (*n* = 1; 118 cm S.L.) and *B. filamentosum* (*n* = 1; 130 cm S.L.) were collected in August and December 1995, respectively, from the whitewater Rio Caquetá, near Araracuara in Colombia (0°37.0′ S; 72°23.112′ W). All otolith preparations were completed at the State University of New York, College of Environmental Science and Forestry (SUNY–ESF) in Syracuse, NY, USA.

Otoliths were first cleaned of organic matter by immersion in a dilute (10% v/v) bleach–water solution followed by gentle abrasion and rinsing in de-ionized water. Further preparation of otoliths for microchemical analyses was adapted from Secor *et al*. [65]. Large lapillar (*B. rousseauxii* and *B. filamentosum*) and sagittal (*P. squamosissimus*) otoliths were cast into circular moulds using EpoFix (Struers) cold-set epoxy. Otoliths were placed in a dehydrator overnight to allow the epoxy to set. Epoxy blocks were sectioned through the core in the transverse plane with a low-speed diamond saw (Buehler, IsoMet) and then polished using progressively finer grades of aluminium oxide lapping paper until the otolith core was exposed (determined by bright-field light microscopy). This process usually involved several repetitions before the core was fully visible. *P. nigricans* otoliths were small enough that they required no cutting and were instead mounted on fused-quartz glass slides using cyanoacrylate adhesive (Loctite) and polished to the core using the methodology described above. Samples were mounted on clean glass or with no backing at all for SXFM analysis, and were mounted on petrographic slides for LA–ICPMS analysis.

Fish ages were also estimated based on previous studies using otoliths, scales and/or length–frequency distributions (table 1). We note, however, that many studies interpreting the ages and/or validating the ages of these fishes remain unpublished or are unavailable. Therefore, we cautiously estimated age for the species herein to provide a broader ecological context for our findings. One notable uncertainty regards the formation of one growth check (annulus) or two growth checks (biannul-) per year. For example, Watson [66] found, via recapture of three individuals, that *Arapaima* sp. scales in Guyana consistently formed one growth mark per year, while Arantes *et al*. [68] verified two marks per year. Silva & Stewart [48] found that *P. nigricans* in Ecuador had annuli, while it appears that the same species may have biannuli in the Central Amazon [69]. For the purposes of this study, we relied on ageing methods developed in the regions from which our specimens came whenever possible. Extensive studies validating otolith ring formation may further elucidate geographical patterns of annuli versus biannuli within and among populations and species. Until such data become available, we must approach our age estimates with a cautious eye.

**Table 1.** Summary of the sources used to age each species, the ageing method used within the study, whether the study reported one growth mark (annulus) or two (biannulus) per year, and whether the study validated the growth rings at age.

species	ageing method	growth mark	literature source	ring validation
*Arapaima* sp.	scales	annulus	Watson 2011^a^^,^^b^ [66]	yes
*Plagioscion squamosissimus*	otoliths	annulus	Loubens 2003 [40]	yes
*Prochilodus nigricans*	otoliths	annulus	Silva & Stewart 2006^a^ [48]	no
*Prochilodus nigricans*	length–frequency	n.a.	Silva 2000^a^^,^^b^ [47]	n.a.
*Brachyplatystoma rousseauxii*	otoliths	biannulus	Alonso & Fabré 2003^b^ [67]	yes
*B. filamentosum*	otoliths	biannulus	Alonso & Fabré 2003^b^^,^^c^ [67]	yes

a Otoliths collected for this study were used in this study.

b Thesis or dissertation.

c Alonso & Fabre [67] studied only *B. rousseauxii*. We applied their methods to *B. filamentosum*.

### 2.2. Sampling with scanning X-ray fluorescence microscopy

The SXFM was conducted at the F3 Beamline Station at the Cornell High Energy Synchrotron Source (CHESS). Samples were taped to cardboard slide frames, and then clipped in place to secure them for sampling. A double-bounce multilayer monochromator was used to create a 16.1 keV beam with 0.6% bandpass. A single-bounce glass capillary [70,71] focused the X-ray beam to a 25–75 µm (depending on sample size) spot on the sample with a photon flux of approximately 1011 counts per second. At each step, the fluorescence spectrum was integrated for 1 s before moving to the adjacent sample location. Fluorescence X-rays were detected with a Vortex energy-dispersive silicon drift quad (4-in-1) detector fitted with an aluminium foil attenuator to reduce high-intensity calcium fluorescence and increase sensitivity to trace elements. Initial spectral processing consisted of screening for a suite of 25 trace elements. Whereas only Sr, Mn and Ca concentrations exhibited consistent variation among and within fishes, other elements (e.g. Se, Zn) were observed in select taxa.

### 2.3. Sampling with laser ablation–inductively coupled plasma mass spectrometry

The LA–ICPMS was conducted at Baker Laboratory at SUNY–ESF. Elemental map collection (SXFM) was complemented by collection of transect data using a NewWave UP-193 nm solid-state laser (Electro Scientific Industries, Inc.) connected to a PerkinElmer Elan DRC-e inductively coupled plasma mass spectrometer. Ablated material was moved via a helium/nitrogen gas mixture from the laser into the ionization chamber of the mass spectrometer. Operating parameters for the laser were 70% power, 10 Hz, 35–100 µm spot size and 3 µm s^−1^ travel time. NIST-610 [72], USGS standard MACS-3 and an in-house pellet made from ground and pressed Freshwater Drum (*Aplodinotus grunniens*) otoliths [73] were used as standards. One-minute washouts of the mass spectrometer were performed between samples to remove residues from previous samples and to collect background counts. Drift corrections were performed as necessary for each sample session.

### 2.4. Data processing and analyses

Data reduction and processing for SXFM were completed using the program PyMCA [74] and the visualization software ‘Praxes,’ developed at CHESS, to produce two-dimensional elemental maps and spatially explicit numerical output. Data were imported into Microsoft Excel, where elements were normalized against Ca (millimole element per mole Ca [5]). A Python script was used to produce final, coloured two-dimensional maps of element-to-Ca ratios. These maps were overlaid on photographs of corresponding otolith sections to compare otolith growth checks with changes in microchemistry and correlate chemical changes with life-history events.

We used elemental data at the otolith margin to infer water conditions at or near point of capture. To infer migration, elements needed to do more than simply rise and fall on a seasonal basis, as this could simply be attributed to annual variation. Therefore, we inferred migration by identifying elements that behaved differently during different life-history stages (i.e. high X : Ca for several years, followed by low X : Ca for several years). A second criterion was to identify simultaneously alternating elements (i.e. high X : Ca coupled with low Y : Ca, and vice versa).

For sedentary fishes, we were particularly interested in Mn : Ca, as this has been shown to be a marker for hypoxia [22] and acidic waters [75], typical characteristics of the environments in which these fishes were caught. While there are currently no laboratory studies determining that environmental Mn incorporates into otoliths in proportion to the Mn dissolved in surrounding waters, several studies have compared otolith Mn in wild fish to known environmental conditions [73,76–78], and the biogeochemistry of manganese as a redox participant is well understood [79]. Limburg *et al*. [22] demonstrated otolith Mn's usefulness as an indicator of hypoxia and anoxia. Blackwater lakes and flooded forests in the Amazon are known to create acidified, hypoxic conditions [34] that promote the dissolution of particulate Mn. We used a threshold of 2.2 × 10^−5^ Mn : Ca as the marker of hypoxic waters following Limburg *et al*. [22]. Chemical markers were expected to remain relatively constant (allowing for slight yearly fluctuations) assuming fishes were indeed sedentary.

To infer migration of juvenile floodplain migrants, Mn : Ca was also of interest. Given the life history of these fishes (spawning in white water, then dispersing into the floodplains and often black water), we expected to find low or undetectable Mn : Ca in the juvenile stage. When they migrate into black water to join adult fishes, we expected to see a marked rise in Mn : Ca. We used the same threshold of 2.2 × 10^−5^ Mn : Ca to infer entrance into black water.

To infer estuary use in migratory catfishes, we were particularly interested in the co-behaviour of Sr and Ba, as both of these elements have been used to identify migrations into and out of estuaries [80,81]. A recent study demonstrated the advantage of simultaneous use of Sr : Ca and Ba : Ca to identify migrations of European eels (*Anguilla anguilla*) from marine to estuarine to freshwater environments [82]. Following Tabouret *et al*. [82], migration into the estuary from the river channel was expected to be marked by a rise in Sr : Ca coupled with a drop in Ba : Ca.

Given the dearth of studies on Amazonian fish otolith chemistry, a broad suite of elements was selected to identify additional elements that may also be of interest but have rarely (or not at all) been reported in previous studies. New environmental markers were identified using the criteria previously outlined and comparisons with published environmental data.

A limitation of this study was the absence of water chemistry data available for all elements in Amazonian environments against which to compare otolith chemistry patterns. Campana [5] notes, however, that looking across multiple specimens in order to establish patterns is a robust technique even without environmental data.

## 3. Results

The main results of this study were: (i) otolith data from *Arapaima* sp. were consistent with a ‘sedentary’ life history involving no long-distance migrations, while the *Plagioscion* otolith bore markers indicating migration among chemically distinct environments; (ii) the majority of sampled *Prochilodus* bore markers consistent with migration from white water to black water; and (iii) *B. rousseauxii* otoliths from both sampled locations had chemical markers consistent with migration into and residency in the Amazon estuary, while *B. filamentosum* lacked these markers. We also found evidence of pollution exposure in two *Prochilodus* individuals as well as a possible new marker for Andean headwaters in *Brachyplatystoma* species.

### 3.1. Sedentary fishes

**Figure 1.**
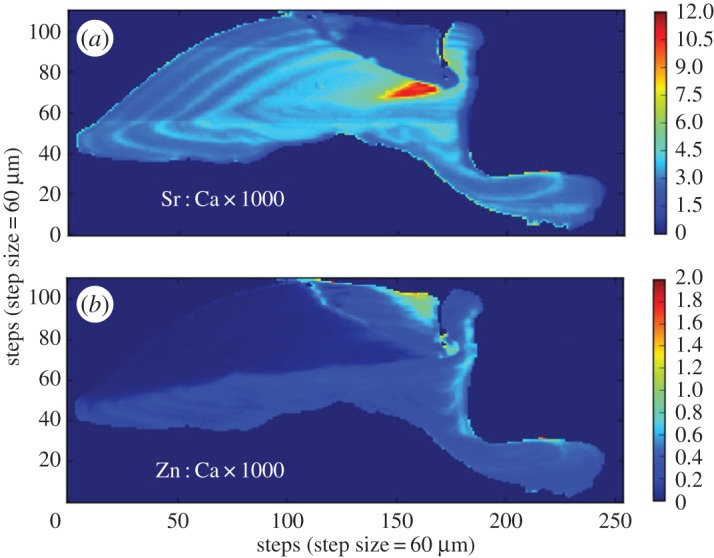
Otolith from Guyana-caught *Arapaima* sp. (*a*) Sr : Ca map; (*b*) Zn : Ca map.

Our estimated age for the *Arapaima* sp. used in this study is 5 years. Sr : Ca in the *Arapaima* otolith showed repeated, annual banding, though a large area of discontinuity occurred near the sulcal groove (figure 1). By contrast, this Sr-depleted region contained elevated Zn, an element that appeared only in discrete zones in the otolith, suggesting differential crystallization (figure 1).

**Figure 2.**
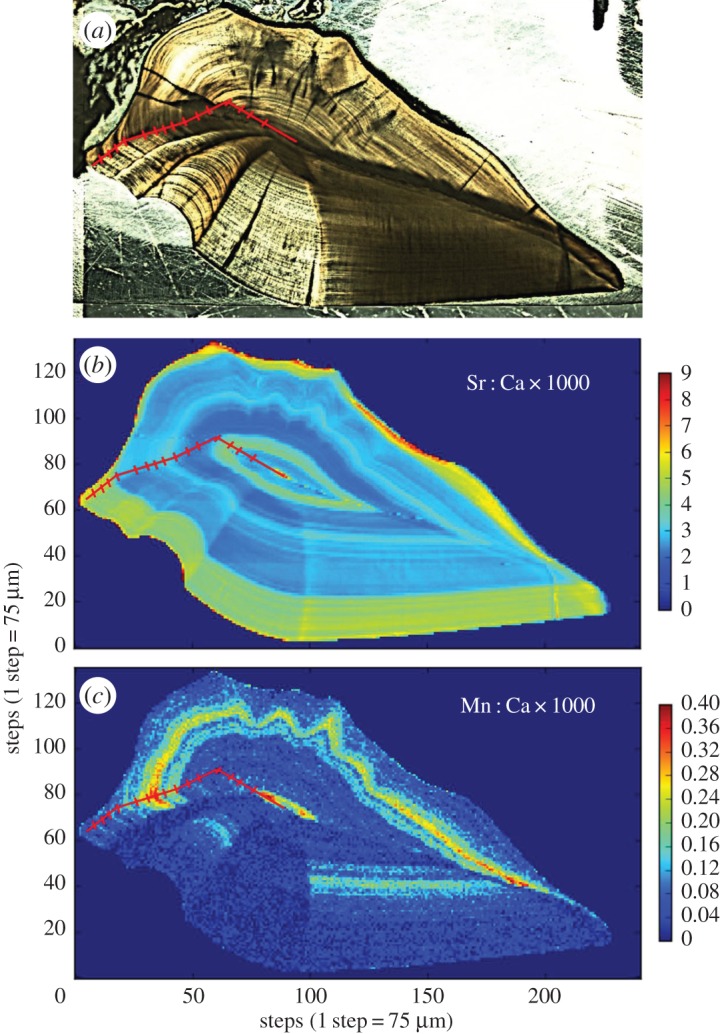
Otolith from Ecuadorian-caught *Plagioscion squamosissimus*. (*a*) Optical image (reflected light); (*b*) Sr : Ca map and (*c*) Mn : Ca map. The red transect marks putative annuli.

We estimated the age of the *P. squamosissimus* to be 15 years. SXFM analysis revealed alternating bands of Sr : Ca and Mn : Ca in this fish that occurred in distinct chemical ‘zones’ (figure 2): thick bands of high and low Sr : Ca alternating with one another every 3–4 years. Bands of elevated Mn : Ca appeared during periods of relatively low Sr : Ca, each zone representing several years. Thin bands of high Mn : Ca co-occurred with the outermost growth checks as well as on the marginal edge of the otolith.

### 3.2. Floodplain migrants

The ages of the *Prochilodus* were difficult to determine using otolith rings. Furthermore, the otoliths used in this study (lapilli) were different from those used in Silva & Stewart [48] (asterisci). Therefore, we divided fish into age classes using the length–frequency data from Silva [47] and Silva & Stewart [48]. We estimated that our samples included four age-0 fish (92–127 mm S.L.), one age-1 fish (197 mm S.L.) and one age-2 fish (267 mm S.L.).

**Figure 3.**
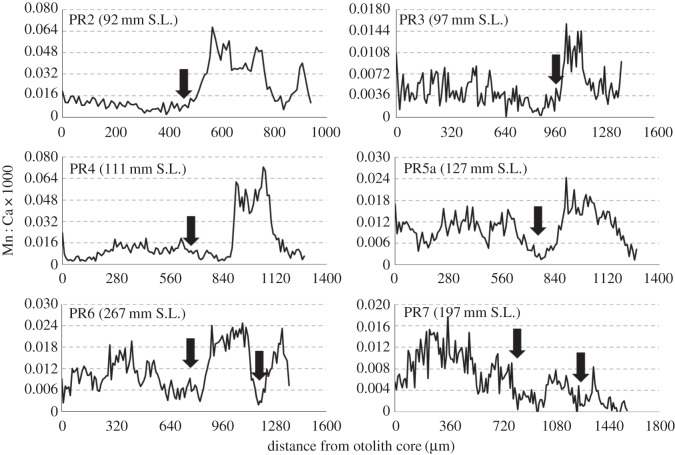
Plots show the variation in Mn : Ca over the lifetime of Ecuadorian-caught *Prochilodus nigricans* measured along a transect from the core of the otolith to its outer edge. Arrows mark the location of growth marks on the otolith. Note that we did not identify these marks as annuli. Different axes are used for each fish to emphasize the patterns of the data.

**Figure 4.**
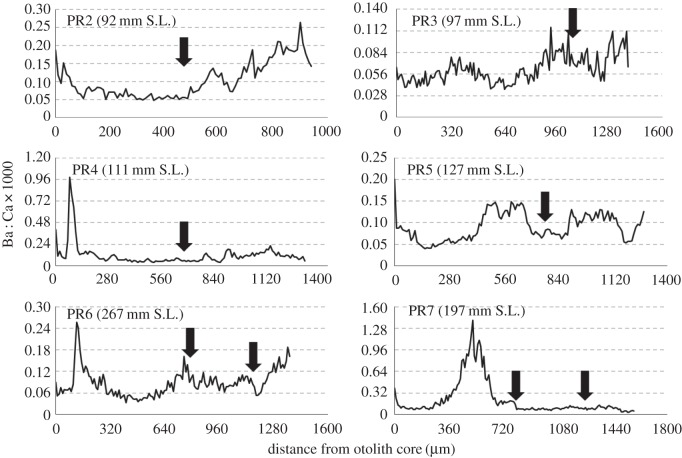
Plots show the variation in Ba : Ca over the lifetime of Ecuadorian-caught *Prochilodus nigricans* measured along a transect from the core of the otolith to its outer edge. Arrows mark the location of growth marks on the otolith. Note that we did not identify these marks as annuli. Different axes are used for each fish to emphasize the patterns of the data.

LA–ICPMS data showed that otoliths of five out of six of our *P. nigricans* specimens recorded a rise, plateau and subsequent drop in Mn : Ca that began about 600–800 µm away from the otolith core (figure 3). Each rise in Mn : Ca was preceded by a visible growth check on the otolith (although not an annulus; figure 3). Two out of six of the *P. nigricans* otoliths also showed a considerable spike in Ba : Ca (figure 4).

### 3.3. Long-distance migrants

**Figure 5.**
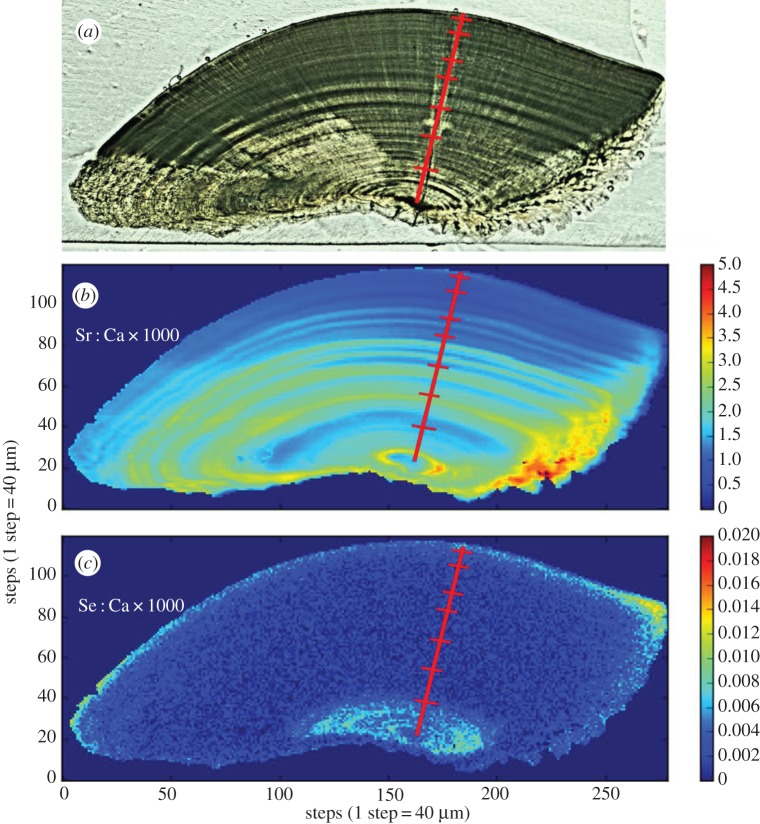
Otolith from Colombian-caught *Brachyplatystoma rousseauxii*. (*a*) Optical image (reflected light). (*b*) Sr : Ca map. (*c*) Se : Ca map. The red transect marks putative biannuli.

**Figure 6.**
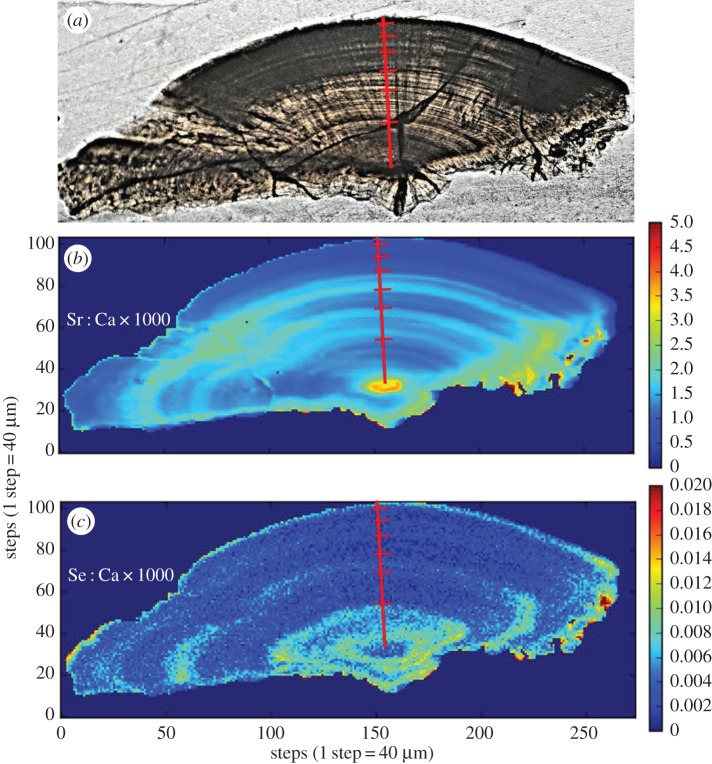
Otolith from Colombian-caught *Brachyplatystoma filamentosum*. (*a*) Optical image (reflected light). (*b*) Sr : Ca map. (*c*) Se : Ca map. The red transect marks putative biannuli.

**Figure 7.**
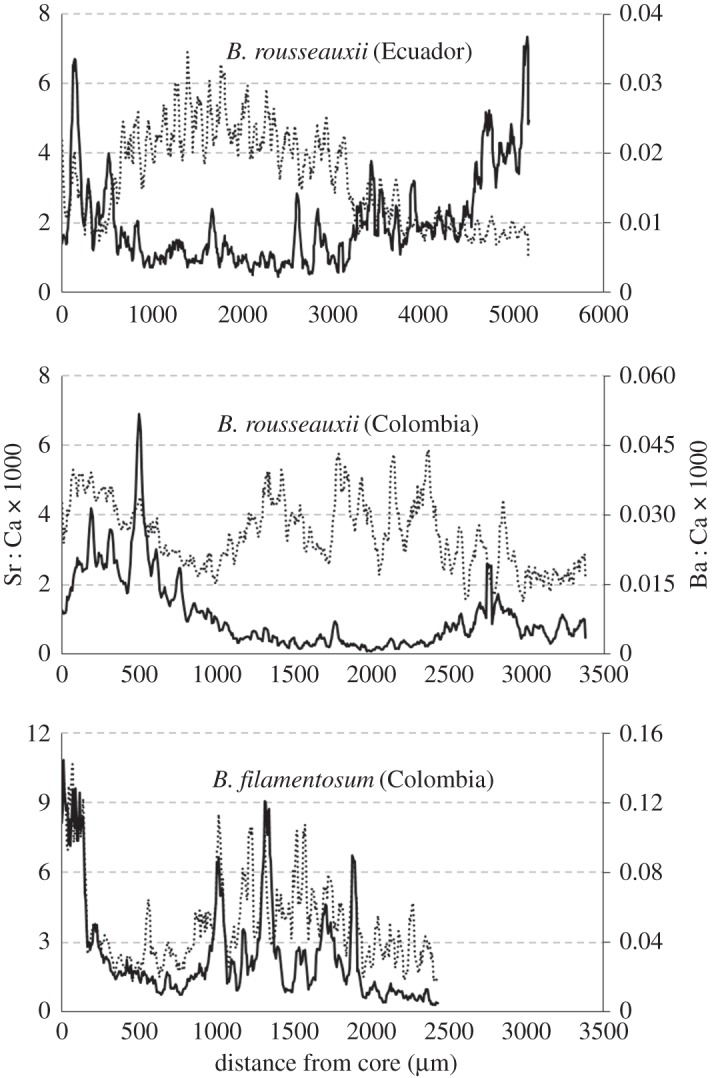
Plots show the variation in Sr : Ca (dashed line) and Ba : Ca (solid line) over the lifetime of two *B. rousseauxii* and one *B. filamentosum*, measured along a transect from the core of the otolith to its outer edge. Different axes are used for each fish to emphasize the patterns of the data. In each graph, the elemental ratio transects were smoothed with 5-point moving averages.

We estimated the ages of the Colombian *B. rousseauxii* and *B. filamentosum* to be 3.5 and 4 years, respectively, and the Ecuadorian *B. rousseauxii* at 3 years following Alonso & Fabré [67]. SXFM revealed alternating bands of Se and Sr in *B. rousseauxii* and *B. filamentosum* that were delineated by biannual growth checks (figures 5 and 6). Marked Se deposition co-occurred with rapid growth in the early life of both *Brachyplatystoma* species, and repeated annually in *B. filamentosum* (figures 5 and 6). LA–ICPMS measurements in both *B. rousseauxii* otoliths showed a decoupling of Sr : Ca and Ba : Ca beginning 500–1000 µm away from the core, in which Sr : Ca rose and plateaued (with annual variation), while Ba : Ca declined and plateaued (figure 7). Around 3000 µm from the core, Sr : Ca declined and Ba : Ca rose. In *B. filamentosum*, Sr : Ca and Ba : Ca rose and declined simultaneously (figure 7). Sr : Ca in both *B. rousseauxii* specimens indicated two distinct life-history phases: one marked by high, variable Sr : Ca during the first 1.5–2 years of life followed by a phase marked by lower, less variable Sr : Ca (figures 5 and 7).

## 4. Discussion

This study offers new insights into South American fish life history and contributes to the growing field of Amazonian fish otolith microchemistry. Using LA–ICPMS and SXFM in combination provided the flexibility to sample many elements simultaneously, which is particularly important in poorly studied systems where it is largely unknown which elements may be useful environmental markers and which may not. This study was not only simple and cost-effective, but also demonstrated how powerful these analytical techniques can be for studying imperilled systems. With a limited sample size, we identified potential new markers for critical habitats and elucidated the life-history complexity of several fish species, while avoiding the issues associated with tagging–tracking methods often used in life-history studies. This study lays the groundwork for a potentially very productive line of research that could do much to provide the necessary biological and ecological basis for conserving these important commercial fishes.

Our results for *Arapaima* sp. were consistent with previous findings that this fish does not migrate among chemically distinct environments. Castello [39] demonstrated that *Arapaima* in the central Amazon appear to migrate short distances among eight different habitats over the course of a flood cycle as water levels regulate habitat availability. However, these habitats are in relatively close proximity with one another and are expected to have roughly the same chemistry. The Sr : Ca observed in the present study (figure 1) oscillated minimally on an annual basis, which would easily be explained by the seasonal introduction of flood waters into the lake from which this fish was captured. The detected Zn (figure 1), which was unique among the fishes in this study, also fits the known ecology of *Arapaima*, outlined above. Zn has been shown to vary inversely with oxygen concentration and pH in the Orinoco River system [83]. The conditions associated with elevated Zn concentrations are common in environments like the lakes that *Arapaima* inhabit. The lifetime Zn : Ca patterns observed in this fish were similar to those of Sr : Ca, displaying small oscillations that could also be explained by annual inundations of the local environment. However, it is also possible that Zn : Ca is under physiological control. Limburg & Elfman [84] found that Zn : Ca in the otoliths of salmoniform and esociform fishes varied seasonally and correlated positively with growth rate, suggesting an underlying physiological mechanism. Additional studies are required to elucidate this in *Arapaima*. Regardless, the otolith microchemistry of this individual appears to be consistent with what one would expect from a typical sedentary fish that undertakes only short-distance lateral migrations among chemically similar environments.

The distinct chemical ‘zones’ of high/low Sr : Ca and high/low Mn : Ca (figure 2) found in the *Plagioscion* otolith suggest that this individual spent multiple growing seasons in several chemically distinct environments. This fish was caught in the lower reaches of the blackwater Cuyabeno drainage near its confluence with the whitewater Rio Aguarico, both of which are surrounded by many floodplain lakes. Therefore, it seems reasonable to conclude that these environments may have been among those used by this fish. Potential migration among such diverse environments contrasts with the behaviour of *Arapaima* sp., whose otolith microchemistry was best explained by short-distance migrations among chemically similar environments. These results suggest that *Plagioscion* may have a more complex life history than previously thought and, furthermore, may be behaviourally distinct from traditional sedentary fishes (e.g. *Arapaima*). Further studies with more extensive sampling will be necessary to better clarify these life-history details and determine how common these hypothesized behaviours may be within this widespread species.

Mn : Ca was also shown to be potentially useful as a marker for black water, which is a critical environment in the life histories of *Plagioscion* and *Prochilodus*. The Mn : Ca values of the thick Mn : Ca bands found in the *Plagioscion* otolith exceeded the hypoxia threshold of 2.2 × 10^−5^ used in this study [22], indicating a water chemistry consistent with preferred spawning conditions (i.e. low dissolved oxygen) identified by Baumgartner *et al*. [42] and Bialetzki *et al*. [43]. The age-0 *Prochilodus* used in this study were probably born in April 1999 during the spawning season and caught for use by Silva & Stewart [48] in September 1999 shortly after having migrated upstream. The otolith growth checks preceding elevated Mn : Ca may mark this migration into black water, where these fishes were caught. Therefore, given that *Prochilodus* spawns in white water, and we found no Mn in strictly whitewater fishes (i.e. both *Brachyplatystoma* species), we hypothesize that the first Mn : Ca peak observed in *Prochilodus* marks the first migration from downstream whitewater nursery habitats to the upstream blackwater rivers.

Sr : Ca and Ba : Ca data suggest that the *B. rousseauxii* individuals used in this study lived their first 1.5–2 years in the Amazon estuary. Using only Sr : Ca, two life-history stages are clearly visible (i.e. the first 1.5–2 years and then thereafter) in *B. rousseauxii* (figures 5*b* and 7). Sr : Ca in otoliths has been demonstrated to increase proportionally with salinity, reflecting the higher concentrations of Sr relative to Ca found in marine waters [80,85,86], and has been used in many studies as a marker in anadromous fishes to indicate a shift between freshwater and marine environments [87,88], including in other tropical species [89,90]. Furthermore, the Sr concentration found in the Amazonian endmember and its tributaries has been shown to be an order of magnitude lower than that found in marine waters [91,92]. The variation in Sr : Ca that we observed is comparable with the differences seen in studies of diadromous fishes that migrate between freshwater and marine environments (e.g. [82,85,88,93,94]). The observed Ba : Ca patterns were also consistent with the migration of a fish from freshwater into an estuary. Elsdon & Gillanders [81] found that otoliths from black bream *Acanthopagrus butcheri* migrating between freshwater rivers and saltwater estuaries had roughly double the otolith Ba : Ca while in freshwater versus while in the estuary, which reflected ambient water chemistry. Recent studies have also shown that Sr and Ba can be used in conjunction to demonstrate the transition between freshwater rivers and estuaries [82,95,96]. For example, the mirrored behaviours of otolith Ba : Ca and Sr : Ca in *B. rousseauxii* (figures 5 and 7) were nearly identical to those observed in European eels [82] and barramundi *Lates calcarifer* [95,96] migrating from freshwater into an estuary. Prior studies using this technique have identified estuary use by fish species with distinctly marine life-history stages, such as hilsa *Tenualosa ilisha* (e.g. [89]) and barramundi (e.g. [96]). However, *Brachyplatystoma* species are thought to remain in freshwater parts of the estuary [27]. If verified by additional studies, these results would mark the first time that this technique has been used to identify estuary use in a tropical migratory fish that lacks a defined marine life-history stage. Therefore, this technique would stand as a powerful tool for identifying residence in this critically important environment.

While the *B. rousseauxii* results were consistent with those of fishes migrating into an estuary from a river, our data suggested that *B. filamentosum* did not migrate passively or actively into the estuary. Rather, Sr : Ca and Ba : Ca for *B. filamentosum* rose and declined simultaneously (figure 7). These results are consistent with those of Hegg *et al*. [17], suggesting that some *Brachyplatystoma* species use the estuary as a nursery area, while others may never reach the estuary and therefore have different life-history strategies.

Our data also indicate that Se may be a useful marker for identifying migration into or out of Andean headwaters. There are few published studies on Se in otoliths and most use it to identify severely polluted environments (e.g. [13,97,98]). However, Yee *et al*. [99] measured Se concentrations in tributaries of the Orinoco Basin, finding that concentrations were highest in tributaries originating in the Andes Mountains (white water), low in the main channel and very low in other tributaries such as those draining the Guyana shield. Because the whitewater tributaries of the Amazon also originate in the Andes, the Amazon and Orinoco Basins probably have a similar pattern of Se concentrations among tributaries. Se : Ca in the *B. rousseauxii* from Colombia was high during roughly the first few months of life, but zero during its assumed residence in the estuary, up until when it was caught in the Rio Caqueta (an Andean tributary), where we found Se : Ca again at the otolith margin (figure 6). The repeated and alternating banding of high Sr : Ca/Ba : Ca and Se : Ca therefore suggest that the Colombian *B. filamentosum* in this study may have migrated into and out of the same or similar headwater tributaries annually during its lifetime (figures 6 and 7), but did not establish residence in those headwater areas.

We hypothesize that the very high Ba : Ca peaks present in two *Prochilodus* specimens were the result of environmental contamination. These values were an order of magnitude higher than those observed anywhere else in the otolith (figure 4) and were the highest concentrations ever observed by one of us (K.E.L.). Both left and right otoliths were analysed on one individual to verify this result. Given that these fish were caught in the Ecuadorian Amazon just downstream from the Lago Agrio oil field, pollution seems a possible cause. Ecuador has a history of poor oil field management since oil exploitation began in 1972, leading to environmental and public health concerns such as increased cancer risk and birth defects among people living nearby [62,100–104]. Measured Ba concentrations from drilling wells at Lago Agrio were as high as 10 100 mg kg^−1^ due to barite contamination; even so-called remediated wells had concentrations over 1000 mg kg^−1^ [105]. Barite (BaSO_4_) is a common additive to ‘drilling mud’, the lubricant used in oil drilling processes [106], and is known to be bioavailable in marine systems, particularly to detritivores [107]. Although largely insoluble near neutral pH, barite acts as a source of reducible sulfate for sulfate-reducing bacteria [108,109], a process that releases Ba into pore waters [110]. Neff [107] also suggested that a fish's stomach may provide a sufficiently acidic environment for dissolution. Further study of the fishes in this region is needed to better describe this phenomenon.

## 5. Conclusion

The data and inferences on the life histories of five Neotropical freshwater fishes reported here provide a foundation upon which to develop testable hypotheses, thereby setting priorities and directions for future studies. Extensive water sampling, both spatially and temporally, will be critical to accurately verify and recreate the migratory strategies hypothesized herein. Ideal future studies should include water sampling at a scale relevant to the species in question (i.e. basin-wide for *Brachyplatystoma*, or regionally for *Prochilodus*). Furthermore, a comparative, basin-wide approach to fish sampling should be taken to elucidate differences among distinct populations (e.g. Ecuadorian versus central Amazonian *Prochilodus*) and/or species (e.g. *Brachyplatystoma* species). Finally, the mechanisms by which newly identified chemical markers were incorporated into otoliths need to be better understood via laboratory experiments and comparison of Amazonian water chemistry to otolith chemistry from fishes caught in those same waters. As demonstrated by this study, a combination of LA–ICPMS and SXFM can facilitate unravelling the complex life histories of these fishes. Subsequent larger studies should provide the migration and habitat use data that are fundamental to developing sound management and conservation strategies for these species. There is also considerable potential for applications of these methods to fishes in other large, tropical rivers of the world that have similar emerging threats [111].

## Data accessibility

Our data are deposited at Dryad: http://dx.doi.org/10.5061/dryad.8kd35.
